# Energy spectrum CT index‐based machine learning model predicts the effect of intravenous thrombolysis in lower limbs

**DOI:** 10.1002/acm2.14048

**Published:** 2023-05-30

**Authors:** Rong Liu, Junlin Yang, Hongkun Yin, Qian Wu, Pengxin Yu, Wei Zhang, Chenglong Li, Guohua Fan, Shenghong Ju, Wu Cai

**Affiliations:** ^1^ Department of Radiology The Second Affiliated Hospital of Soochow University Suzhou Jiangsu China; ^2^ Institute of Advanced Research Infervision Medical Technology Co. Beijing China; ^3^ Department of Vascular Surgery The Second Affiliated Hospital of Soochow University Suzhou Jiangsu China; ^4^ Department of Radiology Zhongda Hospital Medical School of Southeast University Nanjing China; ^5^ State Key Laboratory of Radiation Medicine and Protection Soochow University Suzhou China

**Keywords:** energy spectrum CT, intravenous thrombolysis, lower limbs, machine learning, treatment effect prediction

## Abstract

To develop a noninvasive machine learning (ML) model based on energy spectrum computed tomography venography (CTV) indices for preoperatively predicting the effect of intravenous thrombolytic treatment in lower limbs.

A total of 3492 slices containing thrombus regions from 58 veins in lower limbs in a cohort of 18 patients, divided in good and poor thrombolysis prognosis groups, were analyzed. Key indices were selected by univariate analysis and Pearson correlation coefficient test. A support vector machine classifier‐based model was developed through ten‐fold cross validation. Model performance was assessed in terms of discrimination, calibration, and clinical usefulness at both per‐slice and per‐vessel levels. Continuous variables and categorical variables were compared between good and poor thrombolysis prognosis group by Mann‐Whitney *U*‐test and chi‐square test, respectively. A nomogram was built by integrating clinical factors and the energy spectrum CTV index‐based score calculated by the model.

Six indices selected from 192 indices were used to build the predictive model. The ML model achieved area under the curves (AUCs) of 0.838 and 0.767 [95% CI (confidence interval), 0.825–0.850, 0.752–0.781] in the training and validation datasets at the per‐slice level, and the per‐vessel level AUCs were 0.945 and 0.876 (95% CI, 0.852–0.988, 0.763–0.948) in the training and validation datasets, respectively. The nomogram showed better performance with the per‐vessel level AUC, accuracy, sensitivity and specificity, yielding 0.901(95% CI, 0.793–0.964), 86.2%, 87.9% and 84.0% in the validation dataset, respectively. There was no significant difference in the vessel distribution between good and poor thrombolysis prognosis groups (chi‐square test, *p* = 0.671).

The energy spectrum CTV index‐based ML model achieved favorable effectiveness in predicting the outcome of vessel‐level intravenous thrombolysis. A nomogram integrating clinical factors, and risk score calculated by the developed model showed improved performance and had potential to be used as a noninvasive preoperative tool for clinicians.

## 1 INTRODUCTION

Deep vein thrombosis (DVT), a complex disease, strikes millions of people worldwide.^1^ As the population ages, the incidence of DVT is steadily increasing.^2^, ^3^ DVT is the most worrisome of the etiologies of acute leg swelling, and prompt diagnosis and management are essential to minimize the risk of pulmonary embolism (PE) and post‐thrombotic syndrome (PTS).^4^, ^5^

In select patients with extensive acute (<14 days), proximal (including iliac, femoral and popliteal) DVT who have a low risk of bleeding, catheter‐directed thrombolysis (CDT) has been suggested to reduce acute symptoms and post‐thrombotic morbidity if appropriate expertise and resources are available.^6^ Clinicians usually determine the time of onset of a patient's illness based on the patient's signs and symptoms. However, the diagnosis and staging of DVT of the leg can be difficult, with clinical findings and history being unreliable.^7^ Thrombolysis can accelerate DVT lysis, but it can be associated with significant bleeding risks.^8^ Simultaneously, ineffective thrombolytic therapy can lead to increased treatment costs. Therefore, it is necessary to determine the effectiveness of thrombolysis before thrombolysis. This study attempted to extract spectral information using GE Healthcare's (Discovery CT750 HD, GE Healthcare, Princeton, New Jersey, USA) Gemstone Spectral Imaging (GSI), which is a dual‐energy CT (DECT) scan mode and apply it for the effect of thrombolytic treatment prediction using machine learning (ML) tools.

GSI is a novel technology that relies on a single x‐ray source with fast switching between 2 kV settings (40 and 140 keV) at 0.5 ms intervals during a single gantry rotation to generate low‐ and high‐energy x‐ray spectra. These spectra use the x‐ray attenuation information from two different beam energy levels to characterize the chemical composition of tissues.^9^

To the best of our knowledge, there have been no studies concerning the use of energy spectrum CTV for predicting the effect of venous thrombolysis of the lower limbs. The purpose of this study was to develop a noninvasive ML model based on energy spectrum CTV indices for preoperatively predicting the effect of thrombolytic treatment in the lower limbs. If a patient has a poor prediction of preoperative thrombolysis, then the clinician may change the treatment to, for instance, mechanical thrombectomy. This is helpful for the personalized treatment of patients.

## 2 METHODS

### 2.1 Patient enrollment

This study was approved by the ethical committee of our hospital, and the need for written informed consent was waived. Patients with DVT who underwent CDT from February 2015 to September 2019 were retrospectively recruited. The inclusion criteria were as follows: (1) energy spectrum CTV was performed immediately after admission; (2) the thrombus could be displayed clearly by CT imaging without artifacts and could be measured; (3) the thrombus was located in the post‐caval‐popliteal vein; (4) digital subtraction angiography (DSA) was performed, and thrombolysis was clearly shown. The exclusion criteria were as follows: (1) lack of preoperative energy spectrum CTV exams; (2) received any treatment before thrombolysis; (3) low image quality; (4) contraindications with enhanced CT scan. Finally, preoperative energy spectrum CTV images of 58 vessels from 18 patients with DVT were used in this study. The age, gender, and serum D‐dimer level before thrombolysis of the selected patients were also extracted from the electronic medical record system (Table 1).

**TABLE 1:** Analysis of per‐vessel level characteristics.

	Good thrombolysis prognosis (*n* = 25, per‐vessel level)	Poor thrombolysis prognosis (*n* = 33, per‐vessel level)	*p‐value*
Gender			*0.853*
Female	10	14	
Male	15	19	
Age (years, mean ± SD)	42.9 ± 18.9	52.2 ± 17.3	*0.064*
Serum d‐dimer level			*0.084*
Low (<8 mg/mL)	7	13	
Medium (8 ∼ 16 mg/mL)	2	8	
High (>16 mg/mL)	16	12	
Extent of thrombus (mm, mean ± SD)	43.4 ± 36.1	39.8 ± 28.3	*0.680*

Abbreviation: SD, standard error.

The *p*‐value indicates the statistical significance *p* < 0.05.

### 2.2 Energy spectrum CTV acquisition

Energy spectrum CTV examinations were performed by using a GE Discovery CT750 HD CT scanner (GE Healthcare, Princeton, NJ, USA). The CT scanning parameters were as follows: 120 kV tube voltage, 360 mA tube current, 0.6 s tube rotation time, 512 × 512 matrix, scan field of view (SFOV) large body, and 5 mm section thickness. All CT images were reconstructed using a 0.625 mm slice thickness. For the contrast‐enhanced CT scan, patients were injected with 100 mL of iodine (300 mg I/mL) by a pump injector at a rate of 3 mL/s into the antecubital vein. Images were obtained at post‐injection delays of 12 s after the initiation of contrast material injection. The imaging data were reconstructed at a reconstruction thickness and interval of 0.625 mm and transmitted to AW4.6 workstation (GE Healthcare), through which the indices of energy spectrum CTV were acquired.

### 2.3 Effect valuation of intravenous thrombolysis

Inferior vena cava filters (IVCFs) were used to prevent PE (Optease, Cordis, US; Aegisy, Shenzhen Technology Company, Connecticut; Denali, Bard, US; VenaTech B, Braun, Germany). Filters were inserted percutaneously under fluoroscopic control through a femoral vein. A 4F or 5F unifuse infusion catheter (length 20–40 cm, Unifuse Infusion Catheter, Angiodynamics, New York, USA) was then gently placed with the tip embedded in the proximal extent of the thrombus. Thrombolytic drug urokinase was continuously infused at a dose of 4400 units/kg/h for 24 h. Urokinase (UK, Lizhu Pharmacy Corp, Zhuhai, China) was first injected at a bolus dose of 200 000 to 300 000 U and followed by continuously infusions of 400 000 to 1000 000 U/d pumped through the catheter. The dosage of urokinase was adjusted according to the level of fibrinogen measured by daily analysis of blood coagulation function. If the fibrinogen level dropped below 100 mg/dL, we immediately ceased the use of urokinase. Venography was carried out at 24 h and 48 h intervals to evaluate the progress and location of thrombolysis and to adjust the position of the thrombolysis catheter.

The difference between the pre and post lysis thrombus scores divided by the prelysis score resulted in the percentage of thrombolysis, which was classified into three groups: Grade I 50%; Grade II = 50 to 90%, and Grade III = complete thrombolysis.^10^ Lysis Grades II and III (50%) were considered as successful outcomes (marked lysis).

### 2.4 Thrombus region annotation and energy spectrum CTV index extraction

The preoperative energy spectrum CTV images of each patient were retrieved from the Picture Archiving and Communication System (PACS) to a local workstation. The thrombus region on each slice of preoperative energy spectrum CTV scans was manually labeled by an experienced radiologist. By comparing preoperative energy spectrum CTV images, preoperative and postoperative DSA, the intravenous thrombolysis effect on each slice was confirmed. A total of 48 sets of indices were extracted from the thrombus region of each slice, including MONO, Effective‐Z and Material Density. The list of the 48 sets of indices were presented in Supporting Information. In addition, the average (AVG), maximum (MAX), minimum (MIN) and standard deviation (SD) value of each set were also calculated, which results in the total number of 192 indices (48*4) for analysis.

### 2.5 Index selection and model development

To determine the most important features for predicting the effect of venous thrombolysis, as well as avoid over‐fitting and reduce computational complexity, a two‐step strategy was used to select the most important indices. First, univariate analysis was applied for the selection of indices with significant differences, and only the indices with discriminability (the corresponding *p*‐value of the Mann‐Whitney *U*‐test was lower than 0.05) were kept for further selection. Next, Pearson correlation coefficient analysis was performed to further optimize the features by removing the redundant indices. The correlation between each two indices was calculated, and the index with poorer discriminating ability (with a higher *p*‐value in the first stage) was removed from a pair of highly correlated indices (Pearson correlation coefficient higher than 0.85).

Based on the selected indices, the scikit‐learn toolkit was used to build the per‐slice prediction model.^11^ The support vector machine (SVM) model was used for its superior performance, which has been established theoretically and practically.^12^ All selected indices were standardized before model construction. The radial basis function (RBF) was applied as the kernel function, and the optimal estimation of parameters C and gamma was determined by using grid search with cross‐validation.

This study also used ten‐fold cross‐validation to ensure the optimal training and robustness of the predictive model.^13^ The original dataset, including all slice‐level indices, was randomly split into ten subsets with the same proportions of class labels. A single subset was retained as the validation data, whereas the remaining subsets were used as training data at each time point. The cross‐validation procedure was repeated ten times, with each subset used exactly once as validation data and the performance of all the training and validation samples were calculated.

**FIGURE 1:**
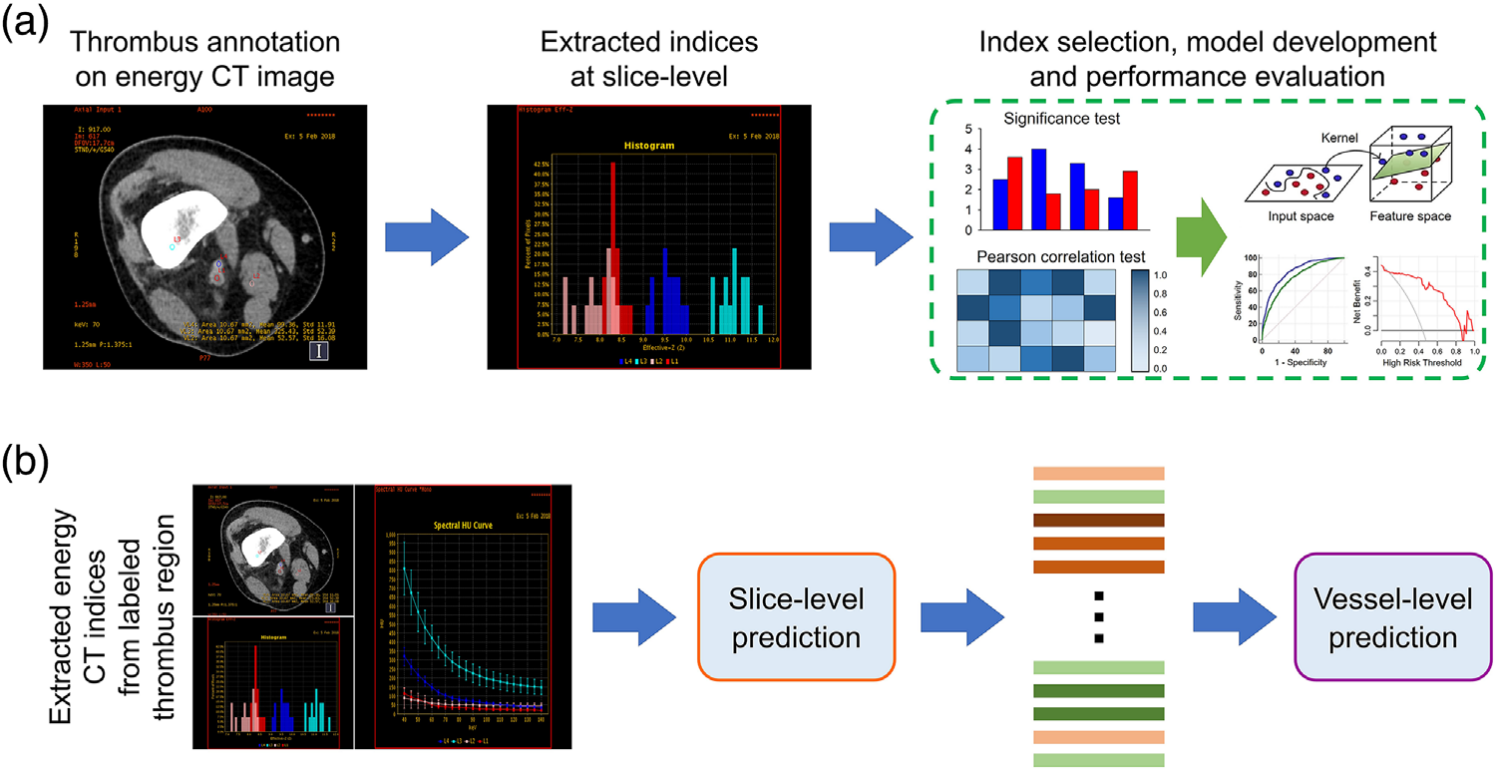
Conceptual architecture of the predictive model. (a) 192 energy spectrum CT indices were automatically extracted from manually‐segmented thrombus region of each slice, and key indices were selected and used as input for support vector machine analysis to predict intravenous thrombolysis probability. (b) The vessel‐level prediction was calculated by averaging the belonging slice‐level probabilities.

As the per‐slice intravenous thrombolysis probability was directly calculated by the predictive model, the vessel‐level prediction of the intravenous thrombolysis effect was assessed by averaging all slice‐level probabilities that belonged to each vessel. The conceptual architecture of the predictive model is shown in Figure 1.

In addition, a nomogram was also developed for clinical application. The clinical factors and the intravenous thrombolysis probability calculated by the per‐vessel model were used as independent predictors. The corresponding parameters of each predictor (coefficient, standard error, wald and *p*‐value) was calculated by using scikit‐learn's via multivariate regression analysis in the training dataset.

The development and validation of the per‐slice and per‐vessel models was performed with InferScholar platform version 3.4 (InferVision).^14^

### 2.6 Model performance evaluation

Receiver operating characteristic (ROC) analysis was performed, and the prediction capability of the per‐slice and per‐vessel models was evaluated by the area under the curve (AUC). An AUC > 0.7 was considered good classification performance.^15^ The accuracy, sensitivity and specificity were calculated under the optimal threshold according to the maximum Youden index.^16^

### 2.7 Calibration analysis and decision curve analysis

The consistency between the actual and predicted thrombolysis probability at the per‐slice or per‐vessel level was assessed by the Hosmer–Lemeshow test and was represented graphically by using 1000 bootstrapping resamples for the evaluation of calibration.^17^ The clinical usefulness of the per‐slice and per‐vessel models was evaluated by decision curve analysis (DCA), and the net benefit was quantified at different threshold probabilities.^18^

### 2.8 Statistical analysis

Continuous variables and categorical variables were compared between the good and poor thrombolysis prognosis groups by the Mann‐Whitney *U*‐test and the chi‐square test, respectively. The calibration curve and the nomogram were plotted using the “rms” package, and the decision curve was plotted using the “rmda” package. The heatmap of selected indices was plotted by using HemI software (version 1.0).^19^ Statistical analyses were performed using SPSS software (version 23.0). Two‐sided *p*‐value less than 0.05 was considered statistically significant.

## 3 RESULTS

### 3.1 Patient characteristics

A total of 20 patients with lower DVT were included. One case was excluded due to low image quality, and one case was excluded due to cavities in the lesion. A total of 58 vessels from 18 patients were finally enrolled in this study, and good thrombolysis prognosis was observed in 25 vessels (four popliteal veins, five superficial femoral veins, four common femoral veins, five external iliac veins, four common iliac veins, and three postcavas), while poor thrombolysis prognosis was observed in 33 vessels (eight popliteal veins, six superficial femoral veins, eight common femoral veins, seven external iliac veins, three common iliac veins, and one postcava).There was no significant difference in the vessel distribution between the good thrombolysis prognosis and poor thrombolysis prognosis groups (chi‐square test, *p* = 0.671). Based on the per‐vessel level, there were no significant differences in gender (*p* = 0.853) or the extent of thrombus (*p* = 0.680) between the good and poor thrombolysis prognosis groups. Although not statistically significant, patients with good thrombolysis prognosis had younger age (*p* = 0.064) and higher serum D‐dimer levels (*p* = 0.084). The detailed clinical characteristics of the patients are shown in Table 1.

### 3.2 Energy spectrum CTV index selection

**FIGURE 2:**
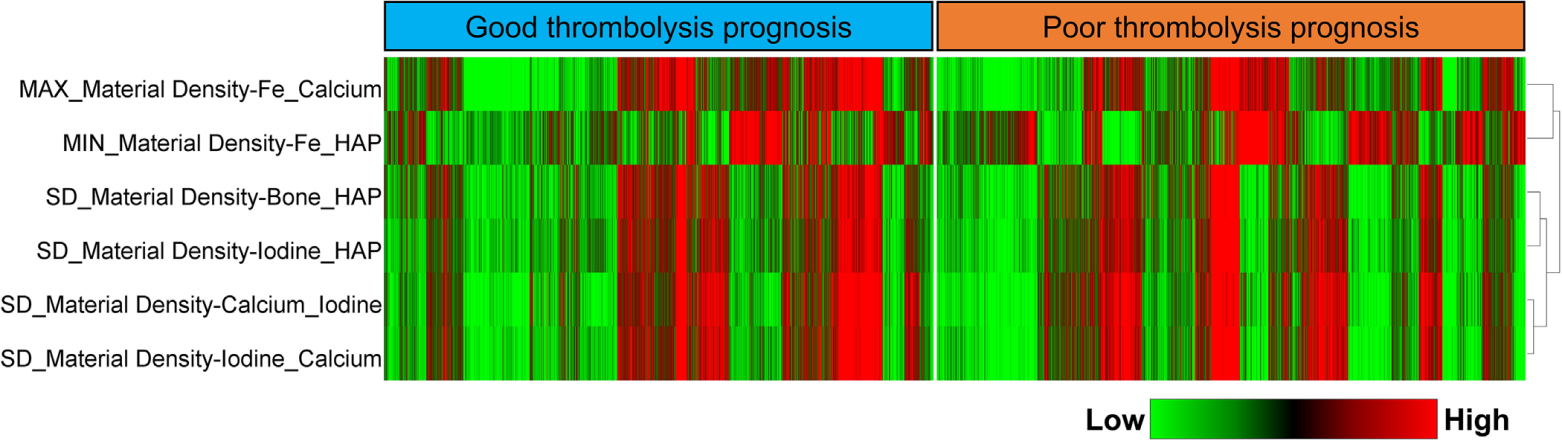
Heatmap analysis of the six selected indices. Each row represents an energy spectrum CT index, and each column corresponds to one slice (separately grouped for the good vs. poor thrombolysis prognosis cohort).

There were 45 indices with significant differences between the good and poor thrombolysis prognosis groups. After Pearson correlation analysis, six material density indices were selected and used for model development. As summarized in Table 2, the differences in these indices between the good and poor thrombolysis prognoses were statistically significant (all *p* values <0.05). The heatmap of the selected indices was plotted according to the standardized index values (Figure 2).

**TABLE 2:** Comparison of the standardized value of the selected energy spectrum CT indexes between the good and poor thrombolysis prognosis groups.

Energy spectrum CT index	Good prognosis	Poor prognosis	*p‐value*
MAX_Material Density‐Fe_Calcium	0.044 ± 1.047	−0.041 ± 0.952	*0.012*
MIN_Material Density‐Fe_HAP	−0.063 ± 0.916	0.058 ± 1.069	*<0.001*
SD_Material Density‐Bone_HAP	0.054 ± 0.912	−0.050 ± 1.073	*0.002*
SD_Material Density‐Calcium_Iodine	0.067 ± 1.039	−0.062 ± 0.958	*<0.001*
SD_Material Density‐Iodine_Calcium	0.080 ± 0.970	−0.074 ± 1.021	*<0.001*
SD_Material Density‐Iodine_HAP	0.036 ± 0.854	−0.033 ± 1.117	*0.042*

The *p*‐value indicates the statistical significance *p* < 0.05.

#### 3.2.1 Development and validation of the predictive model

**FIGURE 3:**
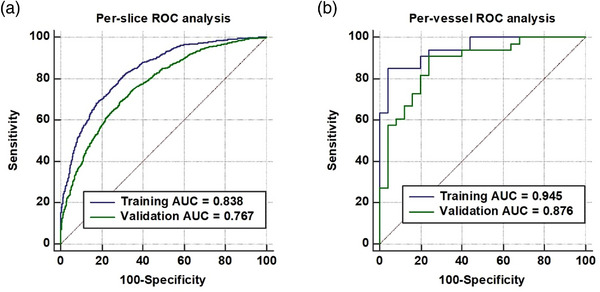
Performance analysis of the predictive model. (a) Per‐slice level ROC curves in the training and validation datasets. (b) Per‐vessel level ROC curves in the training and validation datasets. The 45° dotted line represents the performance of a random classifier. ROC, Receiver operating characteristic.

Model performance was evaluated by ROC analysis with respect to the AUCs in the training and validation datasets. The per‐slice level AUCs of the predictive model were 0.838 (95% CI (confidence interval), 0.825–0.850) in the training dataset and 0.767 (95% CI, 0.752–0.781) in the validation dataset. The model achieved favorable discrimination capability for thrombosis prognosis at the per‐vessel level, with the accuracy and AUC yielding 89.7% and 0.945 (95% CI, 0.852–0.988) in the training dataset and 84.5% and 0.876 (95% CI, 0.763–0.948) in the validation dataset, respectively (Figure 3). The detailed performance of the predictive model at both the per‐slice and per‐vessel levels is presented in Table 3.

**TABLE 3:** Per‐slice and per‐vessel performance of the predictive model.

	Dataset	AUC (95% CI)	Threshold	Accuracy	Sensitivity	Specificity
Per‐slice analysis	Training	0.838 (0.825–0.85)	>0.5016	75.70%	81.20%	69.80%
	Validation	0.767 (0.752–0.78)	>0.5431	69.90%	68.90%	71.10%
Per‐vessel analysis	Training	0.945 (0.852–0.98)	>0.5596	89.70%	84.80%	96.00%
	Validation	0.876 (0.763–0.94)	>0.4951	84.50%	90.90%	76.00%

#### 3.2.2 Calibration and clinical usefulness analysis of the predictive model

**FIGURE 4:**
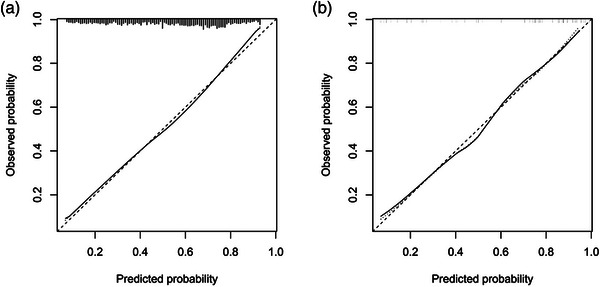
Calibration curve of the predictive model in the validation dataset. (a) Per‐slice level calibration curve. (b) Per‐vessel level calibration curve. The solid line represents the performance of the model without correction for over‐fit. The dotted line is the bootstrap‐corrected performance of the model with a scatter estimate for future accuracy. The graduations visible on the upper portion of the figure represent the distribution of average predicted probabilities of the 1000 bootstrapping resamples. The model showed an almost perfect correlation between the predicted and observed thrombosis prognoses.

**FIGURE 5:**
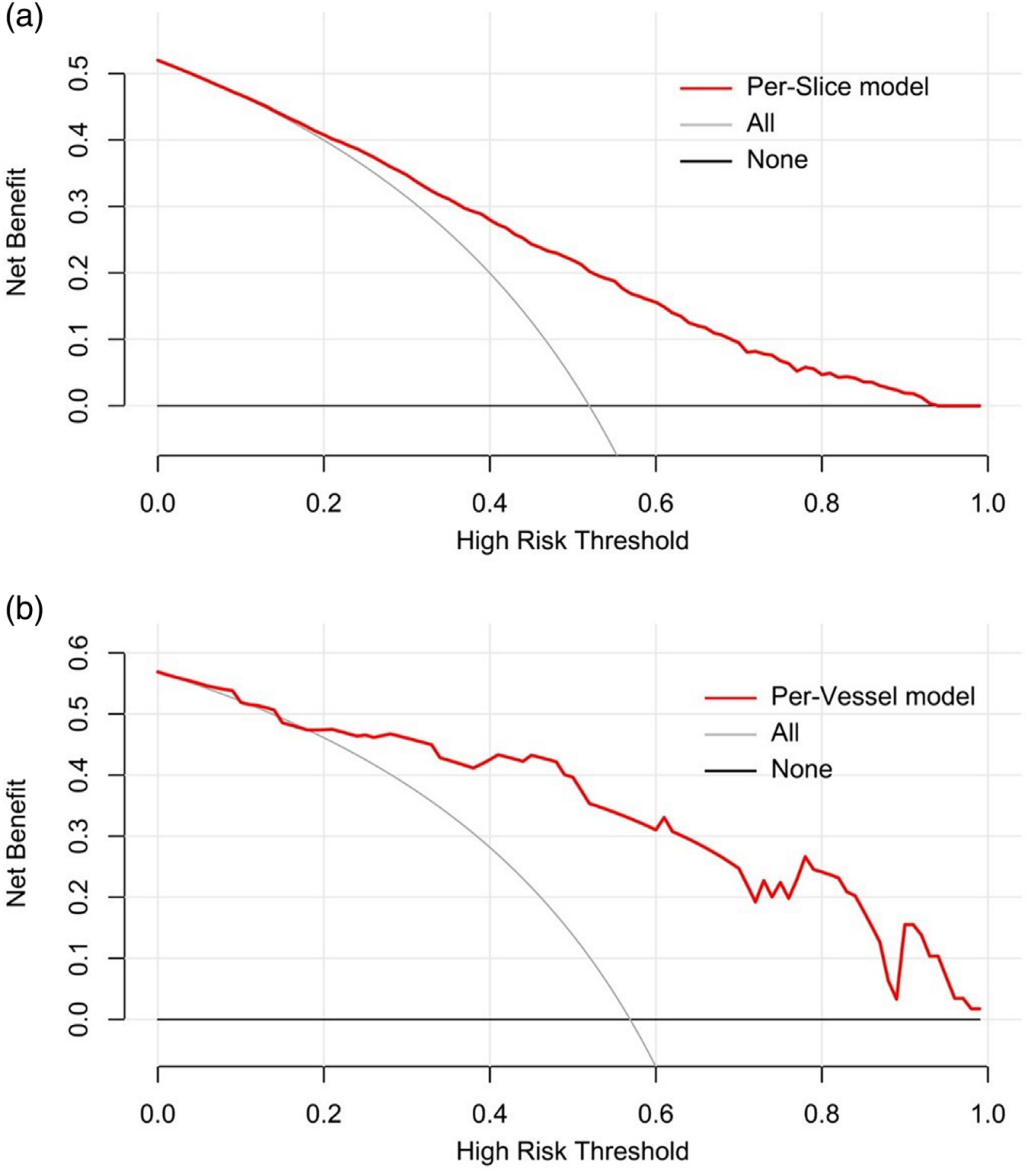
Decision curve analysis of the predictive model at the (a) per‐slice level and (b) per‐vessel level. The red line represents the net benefit of the model across the full range of threshold probabilities. Gray line: all positive, assuming all patients underwent intravenous thrombolysis. Black line: all negative, assuming no patient received intravenous thrombolysis.

Good agreement between the thrombolysis prediction and observation at either the per‐slice level (Hosmer–Lemeshow test, *p* = 0.116) or per‐vessel level (Hosmer–Lemeshow test, *p* = 0.732) in the validation dataset was observed by the calibration curve analysis (Figure 4). The DCA for the predictive model is presented in Figure 5, and the results demonstrated the clinical usefulness of the model at both the per‐slice and per‐vessel levels.

### 3.3 Construction and performance evaluation of the nomogram

**FIGURE 6:**
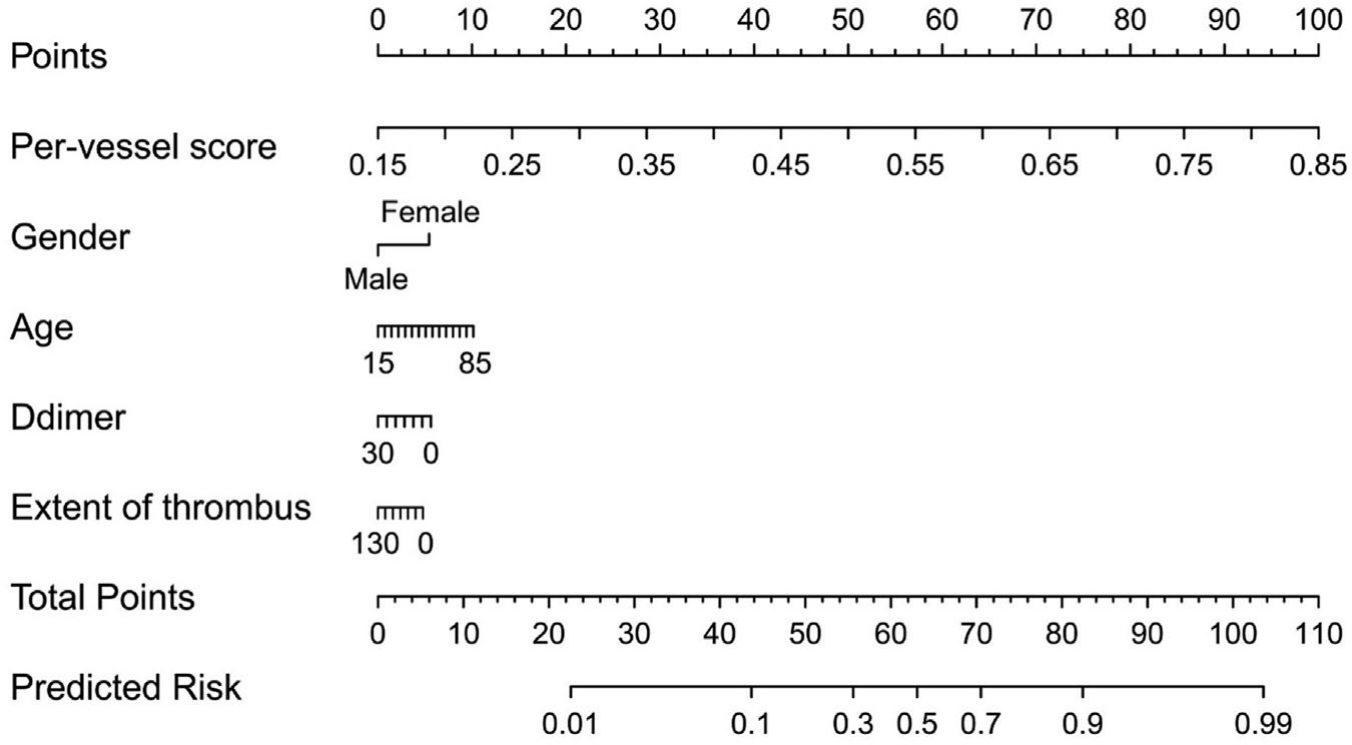
The nomogram integrating age, sex, serum D‐dimer level, extent of thrombus and the per‐vessel level score calculated by the predictive model.

**FIGURE 7:**
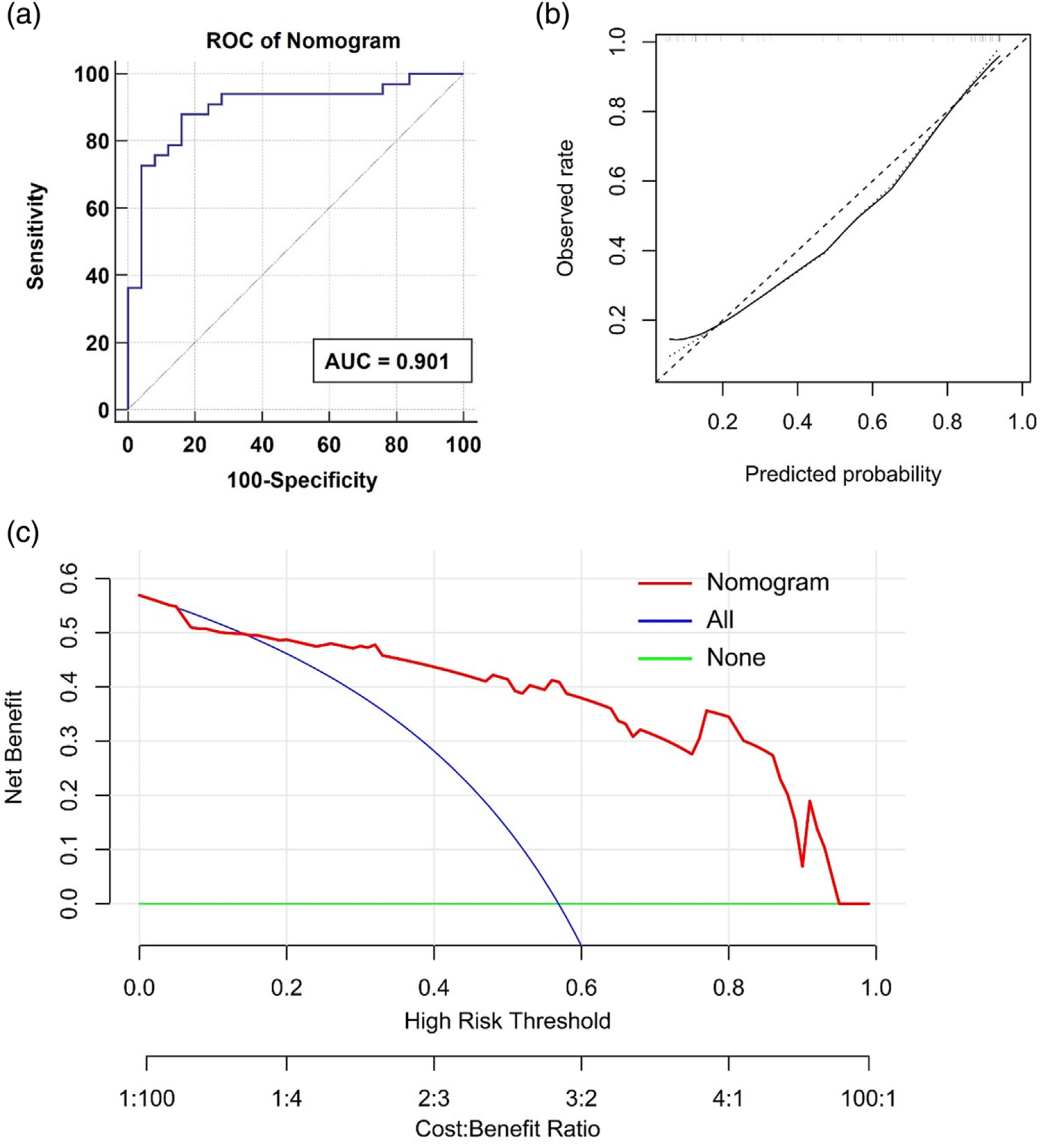
Performance evaluation of the nomogram. (a) ROC analysis of the nomogram in the validation dataset. (b) Calibration curve analysis of the nomogram in the validation dataset. (c) Decision curve analysis of the nomogram at the per‐vessel level. ROC, Receiver operating characteristic.

An easier‐to‐use nomogram was constructed by incorporating age, gender, serum D‐dimer level and the per‐vessel level score (intravenous thrombolysis probability) calculated by the predictive model in the training dataset (Figure 6). The regression coefficients of those variables in the nomogram were summarized in Table 4. The AUC of the nomogram was 0.901 (95% CI, 0.793–0.964), which was higher than that of the predictive model in the validation dataset (*p* = 0.225). The accuracy, sensitivity and specificity of the nomogram under the optimal threshold were 86.2%, 87.9% and 84.0% in the validation dataset, respectively. The nonsignificant statistic of the Hosmer–Lemeshow test for the nomogram (*p* = 0.662) suggested no significant deviation from an ideal fitting. The DCA also demonstrated the clinical usefulness of the nomogram, where the net‐benefit of the nomogram was higher than the “treat all” or “treat none” strategy across almost entire range of the threshold probabilities (Figure 7).

**TABLE 4:** Regression coefficients of the variables in the nomogram.

Variable	Coefficient	Std. Error	Wald	*p*
Per‐vessel score	16.208	4.757	11.609	*<0.001*
Gender	−0.618	0.832	0.553	*0.457*
Age	0.0164	0.0217	0.573	*0.449*
D‐dimer	−0.0214	0.0422	0.256	*0.613*
Extent of thrombus	−0.00331	0.0129	0.0653	*0.798*

The *p*‐value indicates the statistical significance *p* < 0.05.

## 4 DISCUSSION

A large number of quantitative indices can be rapidly extracted and fed into a machine‐learning classifier, which outputs the probability of the effect of CDT. As the indices are automatically extracted by computers, they will be more objective and accurate than subjective and manual measurements. In this study, six material density indices associated with thrombolysis were selected in 192 indices. The results showed that the model achieved favorable discrimination capability for thrombosis prognosis at the per‐vessel level. Therefore, the model has the potential to be a promising energy spectrum imaging tool to preoperatively predict the effect of CDT in the lower limbs, which could facilitate treatment.

Up to now, many scholars attempted to predict the effect of intravenous thrombolysis therapy. Kim et al. and Mfoumou et al. have studied thrombosis hardness by elastography and found that with the aging, the thrombosis hardness increased and the effect of thrombolytic therapy decreased.^20^, ^21^ Yi et al. found that the shear wave velocity (SWV) value has a certain correlation with the thrombolysis effect, and the shorter the time of thrombosis, the better the effect of thrombolytic therapy.^22^ Mewisson et al. found that catheter‐directed infusion of thrombolytic agents had a proven benefit compared to systemic thrombolytic infusion with rapid clot dissolution and reduced thrombolytic dosage.^23^ To the best of our knowledge, this is the first study to predict the CDT effect in lower limbs using an energy spectrum CTV‐based ML model. Prompt DVT treatment can stop the thrombosis process, prevent chronic vein obstruction, maintain valve function, and prevent the development of postthrombosis syndrome.^5^, ^24^ Thrombolytic therapy is an important treatment method for acute deep venous thrombosis of the lower extremities.^25^ Pharmacomechanical thrombolysis can accelerate DVT lysis, but it can be associated with significant bleeding risks, such as gastrointestinal bleeding, cerebral hemorrhage and other important organ hemorrhages.^4^, ^8^, ^26^ So, it is necessary to judge the biological characteristics of thrombi before thrombolytic therapy and predict the curative effect of thrombolysis in advance.

Clinical application research of energy spectrum CT imaging is mainly based on four technical features: hardening artifact removal, image quality and contrast‐to‐noise ratio optimization, quantitative analysis of material and comprehensive analysis of the energy spectrum. Also, energy spectrum CT provides material decomposed images.^27^ The material‐decomposition image can then be used to synthesize the monochromatic image. This GSI scanning mode allows the generation of accurate material decomposed images using various base material pairs to discriminate different materials.^28^

In the present study, 6 indices were selected from 192 for modeling and for the following analysis and speculation, main composed of calcium, HAP, iodine, Fe, as shown in Table 2. Hydroxyapatite (HAP, Ca_10_(PO4)_6_(OH)_2_) is the main mineral composition in human bone. The primary mode of action of hydroxyapatite may act as a calcium phosphate reservoir.^29^ Calcium ions (Ca^2+^) play an important role in blood coagulation. Modarai et al. revealed that the different Ca^2+^ entry mechanisms which exist in platelets are of particular importance for pathological thrombus formation.^30^ Neovascularization is a key component of the re‐canalization of the vein and occurs extensively in venous thrombi.^31^, ^32^ Areas of venous thrombi that contain large numbers of macrophages are also rich in neo‐vascular channels. The thrombus begins to contract and retracts from the vein wall, resulting in the formation of cell‐lined pockets and clefts—new vascular channels between the body of the thrombus and the intima of the vein wall.^31^ All of the patients underwent enhanced CT scans. When a patient has an enhanced CT scan, the iodine contrast agent travels through the bloodstream to new blood vessels in the venous thrombosis. Iron (Fe) is the main component of hemoglobin. Erythroblasts require large amounts of iron for hemoglobin synthesis.^33^ Palmer et al. found that high target hemoglobin levels increased the risk for vascular access thrombosis compared with treatment toward a lower hemoglobin target.^34^ Therefore, there is a conclusive explanation for calcium, HAP, iodine and Fe being in indices.

In this study, the diagnostic effectiveness was evaluated from two aspects (per‐slice and per‐vessel), and the per‐vessel analysis showed higher accuracy, sensitivity and specificity than the per‐slice models. The possible reason was that although the ML model was based on selected indices extracted from slice‐level, it suffered from a high variance from the “highly biased” single slice images which could make the per‐slice model less useful for most practical applications. On the contrast, by aggregating multiple risk scores calculated by the per‐slice model on a series of consecutive slices in one vessel, the per‐vessel model could effectively reduce the variance and therefore achieve better performance.

There are several limitations in this study. First, this was a study from a single center. We will conduct additional studies to avoid bias in modeling and results and to increase the repeatability. Second, the sample size was small. Although the ten‐fold cross‐validation approach was applied to evaluate ML model, a “hold‐out” external dataset is still warranted to test the model's robustness and generalization. Further studies with large sample sizes should be performed to validate the results.

## 5 CONCLUSIONS

The energy spectrum CTV index‐based ML model achieved favorable effectiveness in predicting the outcome of vessel‐level intravenous thrombolysis. A nomogram integrating clinical factors, and risk score calculated by the developed model showed improved performance and had potential to be used as a noninvasive preoperative tool for clinicians.

## AUTHOR CONTRIBUTIONS

Rong Liu and Junlin Yang were involved in drafting the work, acquisition, and analysis and interpretation data for the work. Hong‐kun Yin was involved in analysis and interpretation of data for the work. Qian Wu was involved in revising it critically for important intellectual content. Pengxin Yu was involved in acquisition and analysis data for the work. Wei Zhang was involved in conception or design of the work, revising it critically for important intellectual content. Chenglong Li was involved in Clinical studies; Guohua Fan was involved in acquisition and analysis data for the work; Shenghong Ju was involved in revising it for important intellectual content; Wu Cai was involved in conception or design of the work, acquisition data for the work and final approval of the version to be published.

## CONFLICT OF INTEREST STATEMENT

No authors have any conflict of interest to disclose.

## Supporting information

Supporting information
